# Mediterranean-Style Diet and Other Determinants of Well-Being in Omnivorous, Vegetarian, and Vegan Women

**DOI:** 10.3390/nu15030725

**Published:** 2023-02-01

**Authors:** Joanna Kaluza, Katarzyna Lozynska, Julia Rudzinska, Dominika Granda, Ewa Sicinska, Maria Karolina Szmidt

**Affiliations:** 1 Department of Human Nutrition, Institute of Human Nutrition Sciences, Warsaw University of Life Sciences (WULS-SGGW), Nowoursynowska 166, 02-787 Warsaw, Poland; 2 Department of Nutrition Physiology and Dietetics, Institute of Sport, National Research Institute, Trylogii 2/16, 01-982 Warsaw, Poland

**Keywords:** determinants, Mediterranean diet, vegetarians, vegans, well-being, women

## Abstract

Due to the lack of studies comparing the determinants of well-being in omnivores and vegetarians, we examined associations of socio-demographic and lifestyle factors, including adherence to a Mediterranean-style diet, in relation to well-being in omnivorous, vegetarian, and vegan women. Well-being was assessed using a validated WHO-5 Well-Being Index. Adherence to the Mediterranean-style diet was determined using a modified Mediterranean diet score. The study was conducted on 636 women (23.9 ± 5.7 years), of whom 47.3% were omnivores, 33.2% vegetarians, and 19.5% vegans. The good well-being group (WHO-5 Index ≥ 13 points) comprised 30.9% of the omnivores, 46.0% of the vegetarians, and 57.3% of the vegans. The remaining participants were classified as belonging to the poor well-being group (<13 points). Compared to the omnivores, the vegetarians and vegans had a 1.6-fold (95% CI: 1.04–2.42) and a 2.4-fold (95% CI: 1.45–3.99) higher probability of having good well-being, respectively. In omnivores, the predictors of good well-being were adherence to the Mediterranean-style diet (a 1-score increment was associated with a 17% higher probability of good well-being, P-trend = 0.016), higher self-perceived health status, and lower levels of stress. In vegetarians and vegans, it was older age, higher physical activity (≥3 h/week), 7–8 h sleep time, and similarly to omnivores’ higher self-perceived health status and lower stress level. Our findings indicate that following a Mediterranean-style diet was associated with better well-being in omnivores. Furthermore, we identified that different determinants were associated with well-being in omnivorous and vegetarian and vegan women.

## 1. Introduction

According to the Institute of Health Metrics and Evaluation, in 2019, 970 million people in the world were living with a mental disorder [1]. One year later, mainly due to the COVID-19 pandemic, the World Health Organization (WHO) informed about a 25% increment in the prevalence of anxiety and depression [2]. Depression is one of the largest factors contributing to a global disability, playing a leading role in the global burden of disease [3]. Particular attention is paid to the fact that women are almost two times more likely to be diagnosed with depression and that they are more prone to eating disorders and dissatisfaction with their bodies, which may influence their well-being [3,4,5,6]. The current situation and concerns about further potential increases in the prevalence of mental health diseases result in searching for new solutions to prevent the development of the disease and promote mental well-being.

It is suggested that modifiable factors such as diet, physical activity, stress, and sleep quality may play a role in mental health status [7,8,9]. The role of diet in depression and anxiety risk has been increasingly examined, with promising evidence that adherence to a Mediterranean diet (a dietary pattern based on the traditional diet of people living in the Mediterranean area) may decrease the risk. Results from a meta-analysis (four cohort studies and two cross-sectional studies) indicated that people in the highest versus those in the lowest category of adherence to this diet had a 31% lower risk of incident depressive outcomes [10]. Randomized controlled trials (RCT) on the effect of the Mediterranean diet on depression symptoms are also being conducted, but no results have been published yet [11]. However, depression and anxiety are also diagnosed among vegetarians and vegans; thus, other factors than diet may play an important role [12].

Although in western societies, women are twice as likely as men to be vegan or vegetarian [13], and plant-based diets have been associated with many health benefits (such as decreased incidence of ischemic heart disease and total cancer, lower body mass index, blood glucose, and total cholesterol) [14], an association between vegetarianism and mental health, including well-being, is still unclear. Results from a meta-analysis (2 observational studies, 1 non-RCT, 1 RCT) showed no association between the type of diet followed (omnivores vs. vegetarians and vegans) and well-being [15]. Results regarding depression and the type of diet followed are conflicting. The authors of a meta-analysis (12 observational studies, 1 RCT) showed that vegetarians had a 53% higher risk of depression compared to non-vegetarians [16], whereas results of another meta-analysis indicated no association between following a vegetarian diet and depression nor anxiety [17].

Taking the above into account, in the current study, we hypothesized that higher adherence to the Mediterranean-style diet is associated with better well-being. Moreover, we hypothesized that other factors may determine the well-being of omnivores and of vegetarians and vegans. To address this hypothesis, we investigated the associations between adherence to the Mediterranean-style diet as well as some other lifestyle, health, and socio-demographic determinants in relation to the well-being status in omnivorous as well as vegetarian and vegan women.

## 2. Materials and Methods

### 2.1. Study Design

The cross-sectional study was conducted from January to February 2020 among women from Poland who were following an omnivore or any kind of vegetarian diet. An invitation to take part in the study was published on social media platforms. The study was carried out using the Computer-Assisted Web Interview (CAWI) method. The following inclusion criteria were considered: the female gender, the age of participants from 18 to 50 years, and following an omnivore (no dietary restrictions) or vegetarian (no meat) or vegan diet (no meat and other products from animal sources) longer than 3 months. The criteria of exclusion constituted pregnancy or lactation, following another type than omnivore or vegetarian diet (e.g., low-calorie diet, special diet for medical condition), and following a vegetarian diet for 3 months or less.

The study was conducted according to the guidelines laid down in the Declaration of Helsinki. The study protocol was approved by the Ethics Committee of the Institute of Human Nutrition Sciences of the Warsaw University of Life Sciences (Resolution No. 16/2020). Completion of the administered questionnaire constituted the participants’ informed consent.

### 2.2. Study Population and Data Collection

**Figure 1:**
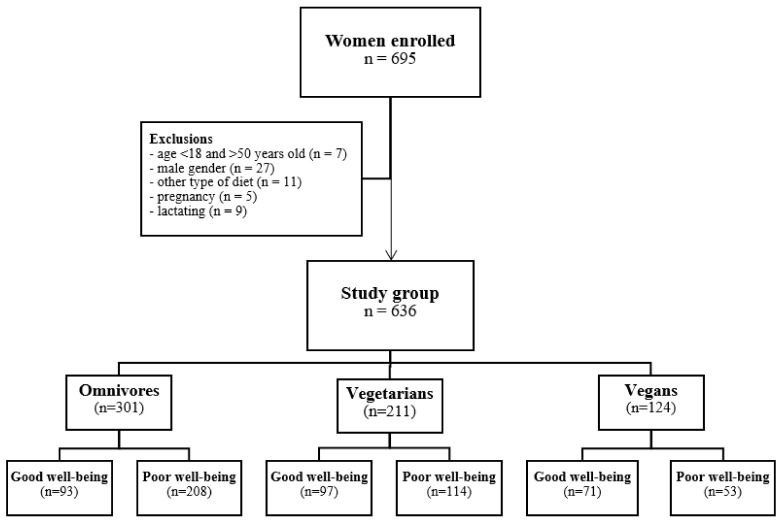
The detailed sampling procedure and recruitment of the studied group.

Out of a total of 695 respondents who filled in the questionnaire, the final analysis included 636 women who met the inclusion criteria (Figure 1). Based on self-reporting of the diet consumed, the participants were classified into three diet groups: omnivorous (n = 301), vegetarian (n = 211), and vegan (n = 124).

The questionnaire included three sections: (1) socio-demographic and lifestyle characteristics, (2) health and well-being status, and (3) a food frequency questionnaire (FFQ).

The socio-demographic section included questions about age, place of residence, education level, and marital status. The lifestyle questions provided data on following a special diet (type, reason, time period), weekly time spent on being physically active, smoking status, the type of dietary supplement usage, as well as average sleeping time. Moreover, the self-reported height and weight of respondents were collected, and the value of the body mass index (BMI) was calculated from the formula: body weight [kg]/height [m]^2^ [18].

Health status questions concerned self-reported health status, the occurrence of chronic diseases, the experience of a traumatic event, and self-perceived stress level using a visual analog scale (1–10 points). Subjective well-being was assessed using the validated World Health Organization Five Well-Being Index (WHO-5 Index), which consists of questions that ask respondents to rate their interest, engagement, and mood [19]. Respondents were asked to rate how much each of the following statements related to their feelings in the past 2 weeks: “I have felt cheerful and in good spirits”, “I have felt calm and relaxed”, “I have felt active and vigorous”, “I woke up feeling fresh and rested”, and “My daily life has been filled with things that interest me”. Respondents rated each item choosing one from six predefined responses (which have been assigned a score): all of the time (5 points), most of the time (4 points), more than half of the time (3 points), less than half of the time (2 points), some of the time (1 point), and at no time (0 points). The WHO-5 Index ranged from 0 (the worst possible well-being) to 25 (the best possible well-being). Women with a score below 13 points were classified into the poor well-being group (according to the official interpretation such values indicate the need for further testing for depression), and those with a score equal to or above 13 points were classified into the good well-being group [19,20].

The FFQ was based on the validated Dietary Habits and Nutrition Beliefs Questionnaire KomPAN [21,22]. The questionnaire was adapted to the study of vegetarians by adding specific food groups such as meat substitutes or dairy substitutes. Food frequency consumption was evaluated in eight categories from “never” to “four times a day or more”, assessing the habitual consumption of food items over the past year. The questionnaire contained the following food groups: vegetables (raw, cooked, canned, baked, etc.); legumes (lentils, beans, chickpeas, peas, soybeans, tofu, tempeh, etc.); fruit (raw, cooked, baked, etc.); wholegrains (coarse grains, brown rice, wholemeal bread, whole grain pasta, oatmeal, etc.); refined grains (e.g., white bread, white rice, pasta, etc.); nuts and seeds; dairy products (milk, yogurt, cheese, etc.); eggs, meat, and meat products (pork, beef, cold cuts, sausages, etc.); fish (cod, salmon, mackerel, herring, etc.); milk substitute (soya drink, almond drink, etc.); meat substitutes (soy cutlets, soy sausages, etc.); fast foods (pizza, burgers, fries, kebabs, chips, etc.); sweets (chocolate, cookies, cakes, candies, donuts, etc.); and alcohol beverages (beers, wines, etc.). For each food group, the categories of frequency of consumption were converted to values that reflected daily frequency consumption.

### 2.3. Assessment of Mediterranean-Style Diet Adherence

To assess the women’s adherence to the Mediterranean-style diet, we used a version of the Mediterranean diet score developed by Trichopoulou et al. [23]. Due to the specificity of vegetarian and vegan diets, we adapted the score to these diets by including dairy and meat plant substitutes of these products, by including wholegrains instead of total cereal, and by replacing the ratio of monosaturated to saturated fatty acids with the use of vitamin D supplements by respondents. After these modifications, the used modified Mediterranean diet score (mMDS) in our study included vegetables, legumes, fruit, nuts and seeds, wholegrains, fish, dairy or dairy substitutes, meat or meat substitutes, vitamin D supplement use, and moderate alcohol consumption (Supplementary Table S1). Consumption of each of the food groups with beneficial health potential (vegetables, legumes, fruit, nuts and seeds, wholegrains, fish) at or above the median was scored as 1 point, and consumption below the median consumption was scored as 0 points. For dairy or dairy substitutes as well as meat or meat substitutes, the reverse scoring was applied. The fact of using vitamin D supplements was scored as 1 point, and not-using as 0 points. For alcohol consumption, 1 point was assigned to women who consumed, on average, between 5 and 25 g of ethanol per day, otherwise 0 points. The total mMED score ranged from 0 (the lack of adherence) to 10 points (the highest adherence to the Mediterranean-style diet).

### 2.4. Statistical Analysis

Descriptive statistics were used to present the percentage distribution of women by the type of diet followed. To establish the relationship between socio-demographic, health, and lifestyle factors, the Chi-square test was used for categorized variables. The normality of continuous variables was tested using the Shapiro–Wilk test. Due to the non-normal distributions of continuous variables, the means in three groups of women were compared using the Kruskal–Wallis test, and the means in two groups were compared using the Mann-Whitney U test.

The associations between women’s well-being and parameters that might constitute its possible predictors were examined separately in omnivores and in vegetarians and vegans. To accomplish this, odds ratios (ORs) with 95% confidence intervals (95% CIs) in two logistic regression models were calculated: the age-adjusted model and the multivariate-adjusted model; with poor well-being as a referent category. The Hosmer–Lemeshow test was used to evaluate the models’ goodness-of-fit.

Multivariate-adjusted models included the following parameters: the age of women (continuous variable), place of residence (urban, rural), education level (primary, high school, or university), marital status (married/having a partner, single/widowed), physical activity (<3, ≥3 h/week), body mass index (<18.5, 18.5–24.9, or ≥ 25 kg/m^2^), self-reported health status (average/poor, at least good), cigarette smoking (no, yes), average sleeping time (≤6, 7–8, or ≥9 h per day), experience a traumatic event (no, yes), stress level (≤4, 5–7, or 8–10 points in VAS), and the mMDS (≤4, 5–6, or ≥7 points). Furthermore, the comprehensive analysis which included in the same multivariate-adjusted model all the above-mentioned variables and the type of diet followed (omnivores, vegetarians, or vegans) was conducted with omnivores in the lowest category of the mMDS (≤4 points) as a reference group.

All statistical analyses were performed using the STATISTICA 13.0 software, and the statistical significance level was set at a *p*-value ≤ 0.05.

## 3. Results

### 3.1. Basic Characteristics and Well-Being

The study was conducted on a group of 636 women (mean aged 23.9 ± 5.7 years), of whom 47.3% followed an omnivore diet, 33.2% a vegetarian diet, and 19.5% had a vegan diet. The basic characteristics of women by type of diet followed are presented in Table 1.

Among vegetarians and vegans, compared with omnivores, a higher percentage of women lived in an urban area, and spent ≥3 h/week being physically active, but fewer of them assessed their health status as at least good. Moreover, a lower percentage of vegans than omnivores and vegetarians declared experiencing a traumatic event in the past.

**Figure 2:**
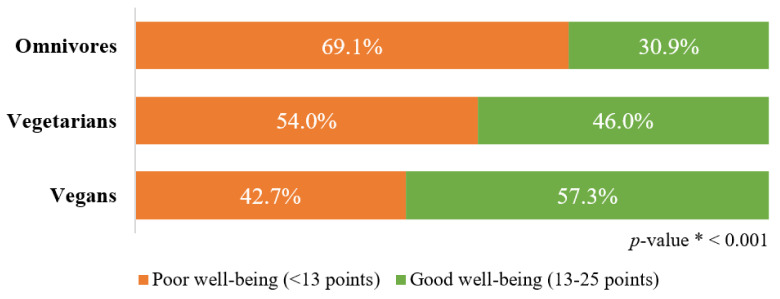
Percentage distribution of omnivores, vegetarians, and vegans by well-being status. Well-being was assessed using the WHO-5 Well-Being Index (range 0–25 points), where < 13 points mean poor well-being and ≥ means good well-being; * *p*-value was determined using Chi-square test.

There was a statistically significant difference between the omnivores, vegetarians, and vegans in regards to their well-being. Women who followed the omnivore diet (10.2 ± 4.2 points) had the lowest mean of the WHO-5 Index, then followed by vegetarians (11.9 ± 4.7 points). Those who followed the vegan diet (13.3 ± 5.1 points) had the highest mean of the WHO-5 Index. Based on the WHO-5 Index, 30.9% of omnivorous women, 46.0% of vegetarians, and 57.3% of vegans were classified into the good well-being group (WHO-5 Index ≥ 13 points), whereas 69.1%, 54.0%, and 42.7%, respectively, were classified into the poor well-being group (WHO-5 Index < 13 points)—Figure 2.

### 3.2. Mediterranean-Style Diet Adherence

**Figure 3:**
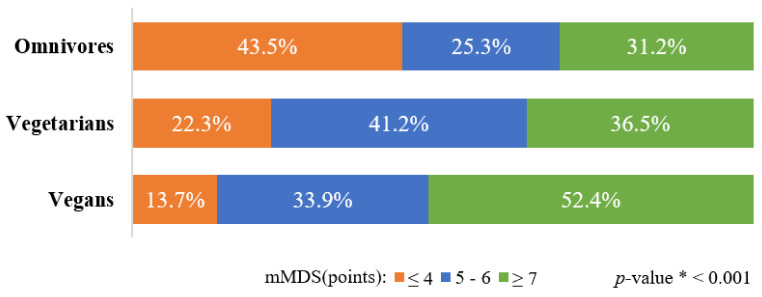
Percentage distribution of omnivores, vegetarians, and vegans by categories of modified Mediterranean diet score (mMDS). * *p*-value was determined using Chi-square test; mMDS (range 0–10 points).

Adherence to the Mediterranean-style diet differed by type of diet followed (Table 2). As expected, the omnivores had the lowest mean of the mMDS (5.1 ± 2.3 points), while the vegans had the highest value of the score (6.3 ± 1.3 points). The percentage distribution of the omnivores, vegetarians, and vegans by category of the mMDS is presented in Figure 3 The lowest adherence to the Mediterranean-style diet (mMDS ≤ 4 points) concerned 43.5% of omnivorous women, 22.3% of vegetarians, and 13.7% of vegans, whereas the highest adherence to this type of diet (mMDS ≥ 7 points) was observed in 31.2%, 36.5%, and 52.4% of women, respectively.

For specific mMDS components, consumption of products beneficial for health, such as vegetables, fruits, legumes, nuts and seeds, wholegrains, and vitamin D supplements, was higher in vegans and vegetarians than in omnivores (Table 2). The consumption of dairy products or dairy substitutes was higher in vegetarians versus omnivores and vegans, while the consumption of meat or meat substitutes was the lowest in vegetarians, then in vegans, and the highest in omnivores. A higher percentage of vegetarians and vegans compared to omnivores met the criteria for mMDS components (except fish and dairy or dairy substitutes; Supplementary Table S1).

### 3.3. Mediterranean-Style Diet Adherence versus Well-Being

The omnivores with good well-being had a statistically higher value of mMDS than those with poor well-being (5.7 ± 2.5 vs. 4.8 ± 2.2 points)—Table 3. They were characterized by more frequent consumption of some Mediterranean-style diet components, that is, vegetables, legumes, fruits, nuts and seeds, and fish. Although the mean value of mMDS did not differ in vegetarians and vegans by well-being status, it was found that vegetarians with good well-being, compared to those with poor well-being, consumed more legumes, wholegrains, and meat substitutes, while vegans with good well-being, compared with those with poor well-being, consumed more fruits, and nuts and seeds.

**Figure 4:**
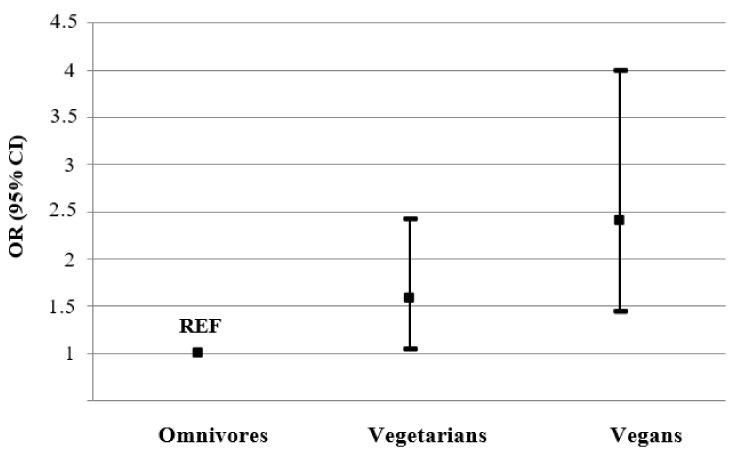
The likelihood of good well-being by the type of diet followed in women. The presented results were adjusted for age, place of living, education, marital status, physical activity, BMI, health status, cigarette smoking, sleeping time, traumatic event, and stress level. Abbreviations: OR—odds ratio; CI—confidence interval; REF—reference group.

Compared to the omnivores, the women on the vegetarian and vegan diets had a 1.6-fold (OR: 1.58, 95% CI: 1.04–2.42) and 2.4-fold (OR: 2.40, 95% CI: 1.45–3.99), respectively, higher probability of having good well-being (Figure 4).

**Figure 5:**
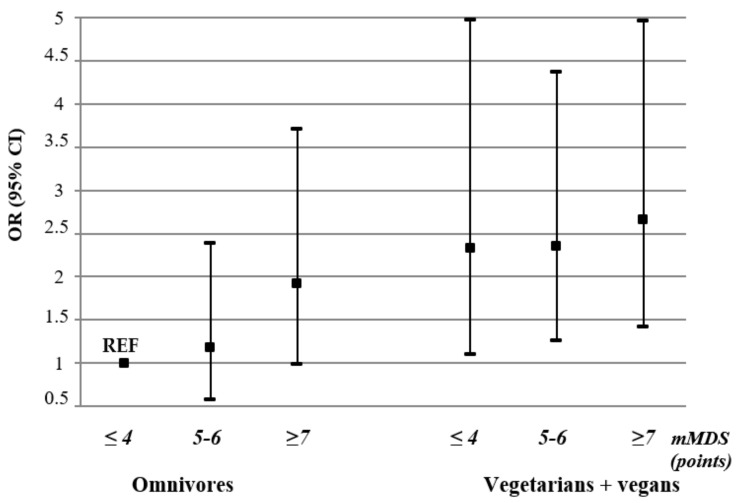
The likelihood of good well-being by the type of diet followed in women and adherence to modified Mediterranean Diet Score (mMDS). The presented results were adjusted for age, place of living, education, marital status, physical activity, BMI, health status, cigarette smoking, sleeping time, traumatic event, and stress level. Abbreviations: OR—odds ratio; CI—confidence interval; REF—reference group.

To examine the associations of the combined effect of the type of diet followed in the context of adherence to the Mediterranean-style diet, we conducted an analysis including the omnivores as well as the vegetarians and vegans in the same model with the omnivores in the lowest category of the mMDS (≤4 points) as a reference group (Figure 5). Compared with the omnivorous women in the lowest category of mMDS, the omnivores in the highest category of the score had a 1.9-fold (OR: 1.92, 95% CI: 1.00–3.71) higher probability of good well-being. The probability of good well-being increased together with increasing adherence to the Mediterranean-style diet in vegetarians and vegans, from 2.3-fold (OR: 2.33, 95% CI: 1.09–4.97) in those in the lowest mMDS category to 2.7-fold (OR: 2.66, 95% CI: 1.42–4.96) in those in the highest category.

### 3.4. Determinants of Women’s Well-Being by Type of Diet Followed

The predictors of women’s well-being by the type of diet followed are presented in Table 4. In the omnivorous group, women who declared at least good health status had a 3.1-fold (OR: 3.11, 95% CI: 1.49–6.49) higher probability of having good well-being compared to those who declared average or poor health status. Furthermore, the omnivores with a low stress level (1–4 points in VAS) compared to those with average or high stress levels (5–7 or 8–10 points, respectively) had significantly lower ratios of odds for good well-being (OR: 0.36, 95% CI: 0.17–0.79 and OR: 0.15, 95% CI: 0.06–0.36, respectively).

Moreover, omnivorous women with the highest adherence to the Mediterranean-style diet (mMDS 7–10 points) were 2.3-fold (OR: 2.33, 95% CI: 1.17–4.64) more likely to have good well-being compared to those with the lowest mMDS (≤4 points). A statistically significant trend between mMDS and well-being was observed; each 1-point increment in the score was associated with a 17% (95% CI: 1–33%; P-trend = 0.016) higher probability of good well-being.

In vegetarians and vegans, some determinants of good well-being such as health status and stress level, overlapped with those determined in omnivores, but some such as age, physical activity level, or sleeping time, differed. A positive statistically significant trend between the women’s age and well-being was observed; each 1 year increment was associated with a 7% (95% CI: 1–12%; P-trend = 0.013) higher probability of good well-being. Vegetarians and vegans who spent ≥3 h/week being physically active were 1.8-fold (OR: 1.81, 95% CI: 1.07–3.05) more likely to have good well-being than those being physically active for <3 h/week. Vegetarian and vegan women who declared at least good health status versus those who declared average or poor health status were more likely to have good well-being (OR: 4.33, 95% CI: 1.86–10.1). Moreover, compared to women who slept 7–8 h, those who slept less (≤6 h/day) had a significantly lower ratio of odds for good well-being (OR: 0.53, 95% CI: 0.29–0.95). In addition, vegetarians and vegans with high stress levels (8–10 points) compared to those with low stress levels (≤4 points) had a significantly lower ratio of odds for good well-being (OR: 0.20, 95% CI: 0.09–0.44).

## 4. Discussion

To our knowledge, this is the first study that comprehensively compared well-being in relation to socio-demographic, health, and lifestyle predictors with particular emphasis on the adherence to a Mediterranean-style diet in omnivores as well as in vegetarians and vegans.

We found that women following the vegetarian and the vegan diet had a higher likelihood of good well-being than the omnivores. This result is in line with the outcomes of some [24,25], but not all [15,26], other studies. In the Seventh Day Adventist study, vegetarians (n = 60, mean age: 45 years) reported significantly less negative emotions than omnivores (n = 78, mean age 41 years) in the Depression Anxiety Stress Scale and the Profile of Mood States questionnaires [24]. Furthermore, in another study conducted by Beezhold et al. [25] (n = 620, aged 25–60 years), it was shown that vegetarians and vegans were less likely to report stress and anxiety than omnivores. Opposite results were obtained by Australian researchers in a cross-sectional study with 9113 female participants aged 22–27 years [26]. It was found that a significantly higher number of vegetarians than non-vegetarians had experienced depression symptoms (29.1% vs. 19.5%, respectively) and deliberate self-harm (10.0% vs. 3.1%, respectively). Nevertheless, it should be noted that the data was collected using closed-ended questions and not with a validated tool. In a meta-analysis that included data from 17,809 individuals, there was no significant association between a vegetarian and a vegan diet and well-being [15]. The authors emphasized that there was a great heterogeneity (in the methods and approaches to defining vegetarianism and veganism) between the studies included, which could explain the lack of association. However, in the same meta-analysis, vegetarians and vegans, compared to omnivores, were at a greater risk for depression (OR = 2.14, 95% CI: 1.11–4.15).

It is plausible that the cause of the inconsistency in the study results on the potential relationship between well-being and a vegetarian and a vegan diet is the effect of its complexity with other well-being determinants. On the one hand, it was proved in both observational [27] and interventional studies [28,29] that a high intake of vegetables and fruit is associated with better well-being, and vegetarians and vegans, compared to omnivores, usually consume more of these products [30]. However, on the other hand, some studies indicated that vegetarians and vegans were more prone to poorer well-being [26] or depression [15,31] and pointed out that this might be due to either an unbalanced diet with micronutrient deficits or social-based reasons. Vegetarians in most parts of the world (Poland included) belong to a social minority that may be associated with experiencing teasing, stereotyping, and sometimes mocking [32,33]. Another possible explanation is that not specifically a vegetarian or a vegan diet causes poorer well-being, but the elimination diet itself [34].

In our study, the mean values of the mMDS and the distribution of women following certain diets by categories of the mMDS differed statistically significantly between omnivores, vegetarians, and vegans. It suggests higher adherence to a Mediterranean-style diet in vegetarians and vegans than in omnivores and is in line with the results of other studies [30,35]. Moreover, in our study, the omnivorous women with higher adherence to the Mediterranean-style diet were less likely to have poorer well-being, whereas such association was not found in vegetarians and vegans. It supports the hypothesis that vegetarians and vegans already followed the Mediterranean-style diet to a great extent (e.g., about a 1.6- and 1.9-times higher percent of vegetarians and vegans, respectively, met the assumption of Mediterranean-style diet criteria for vegetables, 3.1- and 3.5-times for legumes, 1.4- and 1.7-times for fruits, as well as 1.7- and 1.8-times for wholegrains), and their well-being was more influenced by other factors. For example, in our study, vegetarians and vegans with high versus low physical activity (≥3 vs. <3 h/week), as well as those with moderate sleeping time (7–8 vs. ≤ 6 h/day), were more likely to have better well-being, whereas these factors were not associated with well-being in the omnivores.

Positive associations between the adherence to the Mediterranean-style diet and better well-being were described previously in other studies [36,37,38]. Lo Moro et al. [38] in a group of 502 Italian students (76.1% females, median age 23 years) found that the Warwick-Edinburgh Mental Wellbeing scale score gradually increased with higher adherence to the Mediterranean-style diet (*p*-value < 0.001). Also, López-Olivares et al. [36] in a group of 272 Spanish students (64.7% women, mean age 21 ± 4 years) indicated that strict adherence to a Mediterranean-style diet was associated with a positive emotional state assessed via the Positive and Negative Affect Schedule (β = 0.018, *p*-value = 0.009). Better well-being was associated with higher adherence to the Mediterranean-style diet also in 490 Portuguese (71.5% women, mean age 36.5 ± 13.6 years) [37]. The Mediterranean-style diet was also proven to be successful in reducing depression symptoms in interventional studies [39,40]. Due to the high heterogeneity of scales used to evaluate well-being and the adherence to a Mediterranean-style diet, it is difficult to compare different studies’ results; therefore, it seems reasonable to use a unified methodology in future research. The beneficial influence of well-being by higher adherence to the Mediterranean-style diet may result from the high content of antioxidants and dietary fiber, which are typical for this diet type [41,42].

It seems important to mention that, until now, in most studies on the impact of the Mediterranean diet and plant-based diet on well-being or depressive symptoms, women predominate. It is well established that women are characterized by a two-times higher risk of depression [3,6]; the real number of men with depression is not known (they are much less likely to seek help), but it is significant [43]. Thus, it also seems to be important to conduct further research focusing on men since it would be beneficial to establish whether adherence to the Mediterranean diet among vegetarians and vegans has an impact on their well-being.

The main strengths of the current study are its originality as well as a collection of detailed data, which allowed us to comprehensively analyse the associations between lifestyle, health, and socio-demographic factors in relation to subjective well-being status. We used a validated tool in order to assess well-being, and logistic regression multivariable models were implemented to establish well-being determinants. Moreover, to assess Mediterranean-style diet adherence we used a modified version of the Mediterranean Diet Score to adapt it to vegetarians and vegans, that is, including dairy and meat substitutes as well as the use of vitamin D supplements, which seems to be an especially relevant aspect of well-being in vegetarians and vegans. Results of a recently published systematic review showed vitamin D deficiency to be much more prevalent in vegetarians and vegans versus meat-eaters (up to 33% and 67% vs. 6%) [44]. It was also proven previously that vitamin D deficiency is associated with an increased risk of depression [45]. Moreover, this is the first study which assessed the relationship between the adherence to the Mediterranean-style diet and well-being in a group of women from Central Europe.

The current study also has some limitations, among which the most important one is the cross-sectional study design, which makes it impossible to determine cause and effect relationships. Despite using a validated method to assess well-being, such assessment is burdened with a certain degree of subjectivity. Another limitation of our study is the limited number of women who took part in the study, especially vegans, which made it necessary to combine vegetarians and vegans in some analyses because of the statistical power of the tests. Also, younger women were more willing to take part in the study; their overrepresentation limits the possibility of transferring the results to the entire population, especially women over 40. Moreover, due to voluntary participation in the study, women with worse well-being could have been more willing to take part in the study due to their higher interest in the study topic. It resulted in a relatively high percentage of individuals with poor well-being in all groups. Another likely explanation might be the time of data collection; the data was collected in the winter months (January and February) when the chance of sun exposure in Poland is very low. Despite efforts to reliably assess the factors that could be related to well-being, the used questionnaire method to collect data is likely to be affected by some degree of subjectivity as well as some misclassification into subgroups. Moreover, although our FFQ was based on the validated questionnaire [21,22], as in all observational studies, unmeasured or residual confounding cannot be ruled out, and some degree of misclassification in scoring people for the Mediterranean Diet Score components is inevitable.

## 5. Conclusions

The results of this observational study of women indicated that certain predictors may be associated with well-being in omnivores and in vegetarians and vegans. Following the Mediterranean-style diet was associated with better well-being in omnivores, but not in vegetarians and vegans, for whom specific determinants of good well-being were older age, higher physical activity level, and 7–8 h sleep time, together with other predictors which were also present in omnivorous women such as health status and stress level.

A better understanding of the determinants of well-being in omnivores and in vegetarians is relevant for planning public health care to limit increasing problems with mental well-being. Therefore, further comprehensive studies, mainly with an interventional and prospective design, on a significant number of participants who follow different types of diets are needed to identify the risk of poor well-being groups and implement its prevention in men and women.

## Figures and Tables

**Table 1:** Characteristics of women by type of diet followed.

Parameters	Omnivores (n = 301)	Vegetarians (n = 211)	Vegans (n = 124)	*p*-Value *
Age (years)	23.0 ± 4.6 ^a^	24.5 ± 5.9 ^b^	25.2 ± 7.2 ^ab^	0.09
Place of residence, %				<0.001
Urban	73.8	87.2	91.4	
Rural	26.3	12.8	8.1	
Educational level, %				0.07
Primary or high school	56.2	47.9	59.7	
University	43.9	52.1	40.3	
Marital status, %				0.93
Married/partner	48.8	49.8	50.8	
Single/widow	51.2	50.2	49.2	
Physical activity (hours/week), %				
<3	75.1	64.9	53.2	<0.001
≥3	24.9	35.1	46.8	
Body Mass Index (kg/m^2^), %				
<18.5	10.0	10.0	10.5	0.64
18.5–24.9	72.4	76.3	76.6	
≥25	17.9	13.8	12.9	
Health status (self-reported), %				
Average or poor	12.9	18.0	28.2	<0.001
At least good	87.1	82.0	71.8	
Cigarette smoking, %				
No	76.7	68.3	76.7	0.07
Yes	23.2	31.8	23.3	
Sleeping time (hours/day), %				
≤6	27.6	26.5	23.4	0.67
7–8	63.8	64.9	71.0	
≥9	8.6	8.5	5.7	
Experience of a traumatic event, %				
No	67.7	67.8	48.5	<0.001
Yes	32.3	32.2	51.5	
Stress level (VAS, points), %				
≤4	14.0	17.1	21.8	0.38
5–7	45.2	45.5	41.9	
8–10	40.9	37.4	36.3	
WHO-5 Index (points)	10.2 ± 4.2 ^a^	11.9 ± 4.7 ^b^	13.3 ± 5.1 ^c^	<0.001

* *p*-values were calculated using the Kruskal-Wallis test for continuous variable and Pearson’s Chi-square test for categorized variables; ^a,b,c^—different letters indicate statistically significant differences between groups, the Mann-Whitney U test. Abbreviations: WHO-5 Index—World Health Organization (Five) Well-Being Index (0–25 points); VAS—visual analog scale (1–10 points).

**Table 2:** Daily consumption of food groups by type of diet followed (mean ± SD).

Parameters	Omnivores (n = 301)	Vegetarians (n = 211)	Vegans (n = 124)
Modified Mediterranean Diet Score (mMDS), points	5.1 ± 2.3 ^a^	5.6 ± 1.7 ^b^	6.3 ± 1.3 ^c^
Components of mMDS, servings/day			
Vegetables	1.5 ± 1.1 ^a^	2.3 ± 1.1 ^b^	2.6 ± 1.0 ^c^
Legumes	0.1 ± 0.2 ^a^	0.7 ± 0.7 ^b^	1.2 ± 0.8 ^c^
Fruit	1.1 ± 0.9 ^a^	1.4 ± 1.0 ^b^	1.8 ± 1.1 ^c^
Nuts and seeds	0.3 ± 0.5 ^a^	0.6 ± 0.6 ^b^	0.9 ± 0.8 ^c^
Wholegrains	1.0 ± 0.9 ^a^	1.5 ± 1.0 ^b^	1.7 ± 1.0 ^b^
Fish	0.1 ± 0.2	-	-
Dairy or dairy substitutes	0.9 ± 0.9 ^a^	1.1 ± 1.0 ^b^	1.0 ±0.9 ^a^
Meat or meat substitutes	0.9 ± 0.7 ^a^	0.3 ± 0.5 ^b^	0.4 ± 0.5 ^c^
Vitamin D supplements (% of users)	63.1 ^a^	73.0 ^b^	92.0 ^c^
Ethanol intake ^†^	1.0 ± 1.8 ^a^	3.5 ± 13.7 ^b^	3.1 ± 12.8 ^b^

^a,b,c^—different letters indicate statistically significant differences between groups; means were compared using the Mann-Whitney U test, and categorized variable using Pearson’s Chi-square test. ^†^ grams per day.

**Table 3:** Adherence to the Mediterranean-style diet and food consumption by well-being status in omnivores, vegetarians, and vegans (mean ± SD).

Parameters	Omnivores		Vegetarians		Vegans	
	Good Well-Being (n = 93)	Poor Well-Being (n = 208)	Good Well-Being (n = 97)	Poor Well-Being (n = 114)	Good Well-Being (n = 71)	Poor Well-Being (n = 53)
Modified Mediterranean Diet Score (mMDS), points	5.7 ± 2.5 ^a^	4.8 ± 2.2 ^b^	5.8 ± 1.6	5.5 ± 1.8	6.0 ± 1.3	6.2 ± 1.4
Components of mMDS, servings/day						
Vegetables	1.8 ± 1.1 ^a^	1.4 ± 1.1 ^b^	2.4 ± 1.1	2.1 ± 1.1	2.6 ± 1.0	2.5 ± 1.1
Legumes	0.2 ± 0.3 ^a^	0.1 ± 0.2 ^b^	0.9 ± 0.8 ^a^	0.6 ± 0.6 ^b^	1.2 ± 0.8	1.1 ± 0.8
Fruit	1.3 ± 1.0 ^a^	1.0 ± 0.9 ^b^	1.4 ± 1.0	1.4 ± 0.9	1.9 ± 1.0 ^a^	1.6 ± 1.0 ^b^
Nuts and seeds	0.4 ± 0.5 ^a^	0.3 ± 0.5 ^b^	0.6 ± 0.7	0.5 ± 0.5	1.0 ± 0.8 ^a^	0.7 ± 0.6 ^b^
Wholegrains	1.0 ± 1.0	0.9 ± 0.9	1.8 ± 1.0 ^a^	1.3 ± 0.9 ^b^	1.7 ± 1.0	1.6 ± 0.9
Fish	0.2 ± 0.2 ^a^	0.1 ± 0.2 ^b^	-	-	-	-
Dairy or dairy substitutes	0.9 ± 0.8	1.0 ± 1.0	1.3 ± 1.2	1.0 ± 0.9	1.0 ± 0.9	1.0 ± 0.9
Meat or meat substitutes	0.9 ± 0.8	0.8 ± 0.7	0.3 ± 0.5 ^a^	0.2 ± 0.4 ^b^	0.3 ± 0.4	0.4 ± 0.7
Vitamin D supplements (% of users)	49.5	39.9	53.6	45.6	63.4	60.4
Ethanol intake ^†^	0.7 ± 1.0	1.0 ± 2.1	2.7 ± 10.3	4.2 ± 16.1	1.5 ± 2.6	5.3 ± 19.2

^a,b^ different letters indicate statistically significant differences between groups, the Mann-Whitney U test. Good well-being—13–25 points; Poor well-being—<13 points in WHO-5 Well-Being Index. ^†^ grams per day.

**Table 4:** The logistic regression of good well-being by socio-demographic and lifestyle determinants in women.

Study Factors	Omnivores (n = 301)		Vegetarians and Vegans (n = 335)	
	Age-Adjusted OR (95% CI)	Multivariate-Adjusted OR (95% CI)	Age-Adjusted OR (95% CI)	Multivariate-Adjusted OR (95% CI)
Age (years)	1.02 (0.97–1.08)	1.00 (0.93–1.08)	**1.08 (1.04** **–1** **.12)**	**1.07 (1.01** **–1** **.12)**
P for trend	0.390	0.997	<0.001	0.013
Place of residence				
Urban	1.00	1.00	1.00	1.00
Rural	1.17 (0.67–2.03)	1.52 (0.80–2.89)	1.78 (0.85–3.72)	1.52 (0.65–3.57)
Education level				
Primary/high school	1.00	1.00	1.00	1.00
University	1.46 (0.83–2.55)	1.43 (0.76–2.71)	1.07 (0.64–1.78)	0.92 (0.50–1.66)
Marital status				
Married/partner	1.00	1.00	1.00	1.00
Single/widow	0.78 (0.47–1.30)	0.77 (0.43–1.39)	1.32 (0.82–2.10)	1.40 (0.82–2.39)
Physical activity (hours/week)				
<3	1.00	1.00	1.00	1.00
≥3	1.56 (0.90–2.71)	1.20 (0.63–2.29)	**1.88 (1.19–2.97)**	**1.81 (1.07–3.05)**
Body mass index (kg/m^2^)				
<18.5	1.10 (0.49–2.48)	1.43 (0.59–3.49)	**0.43 (0.20–0.96)**	0.54 (0.22–1.31)
18.5–24.9	1.00	1.00	1.00	1.00
≥25	0.71 (0.36–1.41)	0.96 (0.44–2.10)	0.81 (0.42–1.55)	1.03 (0.50–2.13)
P for trend	0.493	0.735	0.524	0.172
Health status (self-reported)				
Average or poor	1.00	1.00	1.00	1.00
At least good	**3.71 (1.89–7.27)**	**3.11 (1.49** **–** **6.49)**	**6.63 (2.99–14.7)**	**4.33 (1.86–10.1)**
Cigarette smoking				
No	1.00	1.00	1.00	1.00
Yes	1.36 (0.77–2.39)	1.50 (0.77–2.90)	0.64 (0.39–1.05)	0.83 (0.47–1.46)
Sleeping time (hours/day)				
≤6	**0.52 (0.28** **–** **0.94)**	0.76 (0.39–1.53)	**0.40 (0.23** **–** **0.68)**	**0.53 (0.29** **–** **0.95)**
7–8	1.00	1.00	1.00	1.00
≥9	0.56 (0.21–1.46)	0.50 (0.18–1.44)	0.51 (0.21–1.21)	0.80 (0.30–2.15)
Experience of a traumatic event				
No	1.00	1.00	1.00	1.00
Yes	0.70 (0.43–1.15)	0.89 (0.50–1.57)	**0.61 (0.38–0.99)**	0.96 (0.55–1.68)
Stress level (VAS, points)				
≤4	1.00	1.00	1.00	1.00
5–7	**0.42 (0.21** **–** **0.86)**	**0.36 (0.17** **–** **0.79)**	**0.45 (0.23–0.89)**	0.55 (0.26–1.13)
8–10	**0.14 (0.07** **–** **0.31)**	**0.15 (0.06** **–** **0.36)**	**0.15 (0.07** **–** **0.30)**	**0.20 (0.09** **–** **0.44)**
P for trend	<0.001	<0.001	<0.001	<0.001
mMDS (points)				
≤4	1.00	1.00	1.00	1.00
5–6	1.60 (0.84–3.03)	1.34 (0.65–2.78)	1.02 (0.55–1.90)	1.03 (0.50–2.11)
≥7	**2.56 (1.43–4.61)**	**2.33 (1.17–4.64)**	1.46 (0.79–2.69)	1.14 (0.56–2.32)
P for trend	0.002	0.016	0.144	0.625

OR—odds ratio; CI—confidence interval; mMDS—modified Mediterranean Diet Score; VAS—visual analog scale. Note: bold font indicates the statistically significant results in the analysis.

## Data Availability

The datasets generated for this study are available on request to the corresponding author.

## Supplementary Materials

The following supporting information can be downloaded at: https://www.mdpi.com/article/10.3390/nu15030725/s1, Table S1: Modified Mediterranean diet score (mMDS) components and scoring criteria (n = 636).

## References

[1] Institute of Health Metrics and Evaluation Global Health Data Exchange (GHDx). https://vizhub.healthdata.org/gbd-results/.

[2] World Health Organization Mental Health and COVID-19: Early Evidence of the Pandemic’s Impact: Scientific Brief, 2 March 2022. https://www.who.int/publications-detail-redirect/WHO-2019-nCoV-Sci_Brief-Mental_health-2022.1.

[3] World Health Organization Depression. https://www.who.int/news-room/fact-sheets/detail/depression.

[4] Lawler M., Nixon E. (2011). Body Dissatisfaction among Adolescent Boys and Girls: The Effects of Body Mass, Peer Appearance Culture and Internalization of Appearance Ideals. J. Youth Adolesc..

[5] Preston C., Ehrsson H.H. (2016). Illusory Obesity Triggers Body Dissatisfaction Responses in the Insula and Anterior Cingulate Cortex. Cereb. Cortex.

[6] Kuehner C. (2017). Why Is Depression More Common among Women than among Men?. Lancet Psychiatry.

[7] Blodgett J.M., Mitchell J.J., Stamatakis E., Chastin S., Hamer M. (2023). Associations between the Composition of Daily Time Spent in Physical Activity, Sedentary Behaviour and Sleep and Risk of Depression: Compositional Data Analyses of the 1970 British Cohort Study. J. Affect. Disord..

[8] Meaklim H., Saunders W.J., Byrne M.L., Junge M.F., Varma P., Finck W.A., Jackson M.L. (2023). Insomnia Is a Key Risk Factor for Persistent Anxiety and Depressive Symptoms: A 12-Month Longitudinal Cohort Study during the COVID-19 Pandemic. J. Affect. Disord..

[9] Yang Y., Liu X., Liu Z.-Z., Tein J.-Y., Jia C.-X. (2023). Life Stress, Insomnia, and Anxiety/Depressive Symptoms in Adolescents: A Three-Wave Longitudinal Study. J. Affect. Disord..

[10] Lassale C., Batty G.D., Baghdadli A., Jacka F., Sánchez-Villegas A., Kivimäki M., Akbaraly T. (2019). Healthy Dietary Indices and Risk of Depressive Outcomes: A Systematic Review and Meta-Analysis of Observational Studies. Mol. Psychiatry.

[11] Bayes J., Schloss J., Sibbritt D. (2021). A Randomised Controlled Trial Assessing the Effect of a Mediterranean Diet on the Symptoms of Depression in Young Men (the ‘AMMEND’ Study): A Study Protocol. Br. J. Nutr..

[12] Berkins S., Schiöth H.B., Rukh G. (2021). Depression and Vegetarians: Association between Dietary Vitamin B6, B12 and Folate Intake and Global and Subcortical Brain Volumes. Nutrients.

[13] Modlinska K., Adamczyk D., Maison D., Pisula W. (2020). Gender Differences in Attitudes to Vegans/Vegetarians and Their Food Preferences, and Their Implications for Promoting Sustainable Dietary Patterns–A Systematic Review. Sustainability.

[14] Dinu M., Abbate R., Gensini G.F., Casini A., Sofi F. (2017). Vegetarian, Vegan Diets and Multiple Health Outcomes: A Systematic Review with Meta-Analysis of Observational Studies. Crit. Rev. Food Sci. Nutr..

[15] Iguacel I., Huybrechts I., Moreno L.A., Michels N. (2021). Vegetarianism and Veganism Compared with Mental Health and Cognitive Outcomes: A Systematic Review and Meta-Analysis. Nutr. Rev..

[16] Ocklenburg S., Borawski J. (2021). Vegetarian Diet and Depression Scores: A Meta-Analysis. J. Affect. Disord..

[17] Askari M., Daneshzad E., Darooghegi Mofrad M., Bellissimo N., Suitor K., Azadbakht L. (2022). Vegetarian Diet and the Risk of Depression, Anxiety, and Stress Symptoms: A Systematic Review and Meta-Analysis of Observational Studies. Crit. Rev. Food Sci. Nutr..

[18] WHO Consultation on Obesity, World Health Organization (2000). Obesity: Preventing and Managing the Global Epidemic: Report of a WHO Consultation.

[19] World Health Organization (1998). Regional Office for Europe Wellbeing Measures in Primary Health Care/the DepCare Project: Report on a WHO Meeting: Stockholm, Sweden, 12–13 February 1998.

[20] Topp C.W., Østergaard S.D., Søndergaard S., Bech P. (2015). The WHO-5 Well-Being Index: A Systematic Review of the Literature. Psychother Psychosom.

[21] Jeżewska-Zychowicz M., Gawęcki J., Wądołowska L., Czarnocińska J., Galiński G., Kołłajtis-Dołowy A., Roszkowski W., Wawrzyniak A., Przybyłowicz K., Krusińska B. (2018). Dietary Habits and Nutrition Beliefs Questionnaire for People, Version 1.1.–Interviewer Administered Questionnaire. Chapter 1. Dietary Habits and Nutrition Beliefs Questionnaire and the Manual for Developing of Nutritional Data.

[22] Kowalkowska J., Wadolowska L., Czarnocinska J., Czlapka-Matyasik M., Galinski G., Jezewska-Zychowicz M., Bronkowska M., Dlugosz A., Loboda D., Wyka J. (2018). Reproducibility of a Questionnaire for Dietary Habits, Lifestyle and Nutrition Knowledge Assessment (KomPAN) in Polish Adolescents and Adults. Nutrients.

[23] Trichopoulou A., Bamia C., Trichopoulos D. (2009). Anatomy of Health Effects of Mediterranean Diet: Greek EPIC Prospective Cohort Study. BMJ.

[24] Beezhold B.L., Johnston C.S., Daigle D.R. (2010). Vegetarian diets are associated with healthy mood states: A cross-sectional study in Seven Day Adventist adults. Nutr. J..

[25] Beezhold B., Radnitz C., Rinne A., DiMatteo J. (2015). Vegans Report Less Stress and Anxiety than Omnivores. Nutr. Neurosci..

[26] Baines S., Powers J., Brown W.J. (2007). How Does the Health and Well-Being of Young Australian Vegetarian and Semi-Vegetarian Women Compare with Non-Vegetarians?. Public Health Nutr..

[27] Głąbska D., Guzek D., Groele B., Gutkowska K. (2020). Fruit and Vegetable Intake and Mental Health in Adults: A Systematic Review. Nutrients.

[28] Conner T.S., Brookie K.L., Carr A.C., Mainvil L.A., Vissers M.C.M. (2017). Let Them Eat Fruit! The Effect of Fruit and Vegetable Consumption on Psychological Well-Being in Young Adults: A Randomized Controlled Trial. PLoS ONE.

[29] Martins L.B., Braga Tibães J.R., Sanches M., Jacka F., Berk M., Teixeira A.L. (2021). Nutrition-Based Interventions for Mood Disorders. Expert Rev. Neurother..

[30] Clarys P., Deliens T., Huybrechts I., Deriemaeker P., Vanaelst B., De Keyzer W., Hebbelinck M., Mullie P. (2014). Comparison of Nutritional Quality of the Vegan, Vegetarian, Semi-Vegetarian, Pesco-Vegetarian and Omnivorous Diet. Nutrients.

[31] Nezlek J.B., Forestell C.A., Newman D.B. (2018). Relationships between Vegetarian Dietary Habits and Daily Well-Being. Ecol. Food Nutr..

[32] Jetten J., Haslam S.A., Cruwys T., Greenaway K.H., Haslam C., Steffens N.K. (2017). Advancing the Social Identity Approach to Health and Well-Being: Progressing the Social Cure Research Agenda. Eur. J. Soc. Psychol..

[33] Bagci S.C., Olgun S. (2019). A Social Identity Needs Perspective to Veg*nism: Associations between Perceived Discrimination and Well-Being among Vegans in Turkey. Appetite.

[34] Matta J., Czernichow S., Kesse-Guyot E., Hoertel N., Limosin F., Goldberg M., Zins M., Lemogne C. (2018). Depressive Symptoms and Vegetarian Diets: Results from the Constances Cohort. Nutrients.

[35] Avital K., Buch A., Hollander I., Brickner T., Goldbourt U. (2020). Adherence to a Mediterranean Diet by Vegetarians and Vegans as Compared to Omnivores. Int. J. Food Sci. Nutr..

[36] López-Olivares M., Mohatar-Barba M., Fernández-Gómez E., Enrique-Mirón C. (2020). Mediterranean Diet and the Emotional Well-Being of Students of the Campus of Melilla (University of Granada). Nutrients.

[37] Andrade V., Jorge R., García-Conesa M.-T., Philippou E., Massaro M., Chervenkov M., Ivanova T., Maksimova V., Smilkov K., Ackova D.G. (2020). Mediterranean Diet Adherence and Subjective Well-Being in a Sample of Portuguese Adults. Nutrients.

[38] Lo Moro G., Corezzi M., Bert F., Buda A., Gualano M.R., Siliquini R. (2021). Mental Health and Adherence to Mediterranean Diet among University Students: An Italian Cross-Sectional Study. J. Am. Coll. Health.

[39] Jacka F.N., O’Neil A., Opie R., Itsiopoulos C., Cotton S., Mohebbi M., Castle D., Dash S., Mihalopoulos C., Chatterton M.L. (2017). A Randomised Controlled Trial of Dietary Improvement for Adults with Major Depression (the ‘SMILES’ Trial). BMC Med..

[40] Parletta N., Zarnowiecki D., Cho J., Wilson A., Bogomolova S., Villani A., Itsiopoulos C., Niyonsenga T., Blunden S., Meyer B. (2019). A Mediterranean-Style Dietary Intervention Supplemented with Fish Oil Improves Diet Quality and Mental Health in People with Depression: A Randomized Controlled Trial (HELFIMED). Nutr Neurosci.

[41] Ventriglio A., Sancassiani F., Contu M.P., Latorre M., Di Slavatore M., Fornaro M., Bhugra D. (2020). Mediterranean Diet and Its Benefits on Health and Mental Health: A Literature Review. Clin. Pract. Epidemiol. Ment. Health.

[42] Gantenbein K.V., Kanaka-Gantenbein C. (2021). Mediterranean Diet as an Antioxidant: The Impact on Metabolic Health and Overall Wellbeing. Nutrients.

[43] Men and Depression. https://www.nimh.nih.gov/health/publications/men-and-depression.

[44] Neufingerl N., Eilander A. (2021). Nutrient Intake and Status in Adults Consuming Plant-Based Diets Compared to Meat-Eaters: A Systematic Review. Nutrients.

[45] Anglin R.E.S., Samaan Z., Walter S.D., McDonald S.D. (2013). Vitamin D Deficiency and Depression in Adults: Systematic Review and Meta-Analysis. Br. J. Psychiatry.

