# Supplementary material for: Mediterranean-Style Diet and Other Determinants of Well-Being in Omnivorous, Vegetarian, and Vegan Women
Source: Nutrients. 2023 Feb 1;15(3):725. doi: 10.3390/nu15030725 (PMC9920499; doi:10.3390/nu15030725)
Supplement: Supplementary file 1 [file nutrients-15-00725-s001.zip › nutrients-2160853-supplementary.pdf]

Table S1. Modified Mediterranean diet score (mMDS) components and scoring criteria (n=636).

**Food group**	**Median (servings/day)**	**Scoring criteria (1 point)**	**Percent of women who met the scoring criteria**		
			**Omnivores**	**Vegetarians**	**Vegans**
Vegetables	2.50	≥ median	44.2	72.5	83.1
Legumes	0.14	≥ median	26.9	84.4	95.2
Fruit	1.00	≥ median	47.8	69.2	81.5
Nuts and seeds	0.14	≥ median	50.2	77.7	85.5
Wholegrains	1.0	≥ median	41.9	70.6	75.8
Fish	0	≥ median	74.8	0	0
Dairy or dairy substitutes	0.79	< median	48.2	39.8	41.9
Meat or meat substitutes	0.43	< median	16.9	72.0	57.3
Vitamin D supplement use	-	yes	42.9	49.3	62.1
Ethanol intake †	-	5 – 25 g/day	5.6	8.5	8.9

†Low: <5 g/day; moderate: ≥5 g/day and ≤25 g/day; high: >25 g/day.
